# Modulating Oxidative Stress in B Cells Promotes Immunotherapy in Food Allergy

**DOI:** 10.1155/2022/3605977

**Published:** 2022-01-21

**Authors:** Hao-Tao Zeng, Yu Liu, Miao Zhao, Jiang-Qi Liu, Qiao-Ruo Jin, Zhi-Qiang Liu, Yan Li, Zhi-Gang Liu, Bai-Sui Feng, Pingchang Yang

**Affiliations:** ^1^Department of Clinical Laboratories, Longgang E.N.T Hospital & Shenzhen Key Laboratory of E.N.T, Institute of E.N.T Shenzhen, China; ^2^The 3rd Affiliated Hospital of Shenzhen University, Shenzhen, China; ^3^Guangdong Provincial Key Laboratory of Regional Immunity and Diseases, Shenzhen, China; ^4^Institute of Allergy & Immunology, Shenzhen University School of Medicine, State Key Laboratory of Respiratory Disease Allergy Division at Shenzhen University, Shenzhen, China; ^5^Department of Gastroenterology, Second Affiliated Hospital, Zhengzhou University, Zhengzhou, China

## Abstract

Allergen-specific immunotherapy (SIT) is the mainstay in the treatment of allergic diseases; its therapeutic efficacy is to be improved. Bacterial flagellin (FGN) has immune regulatory functions. This study investigates the role of FGN in promoting immunotherapy efficacy through modulating oxidative stress in regulatory B cells (Bregs). Blood samples were collected from patients with food allergy (FA) and healthy control (HC) subjects. CD19^+^ CD5^+^ Bregs were purified from blood samples by flow cytometry cell sorting. A murine FA model was developed with ovalbumin as the specific antigen. The results showed that peripheral Bregs from FA patients showed lower TLR5-related signals and higher apoptotic activities. The peripheral Breg frequency was negatively correlated with serum FGN levels in FA patients. Exposure to a specific antigen in culture induced antigen-specific Breg apoptosis that was counteracted by the presence of FGN. FGN diminished specific antigen-induced oxidative stress in Bregs. The STAT3/MAPKp38/NF-*κ*B signal pathway was involved in the FGN/TLR5 signal-promoted superoxide dismutase expression in Bregs. Administration of FGN promotes the SIT efficacy in suppressing experimental FA. In summary, administration of FGN promotes SIT efficacy on FA, suggesting that the combination of FGN and SIT can be a novel therapy that has the translational potential to be employed in the treatment of allergic diseases.

## 1. Introduction

Allergen-specific immunotherapy (SIT) is the mainstay in the treatment of allergic diseases currently. By administration of small doses of specific allergens, regulatory immune cells, such as regulatory T cells (Tregs) and regulatory B cells (Bregs), are generated to suppress the aberrant immune responses and thus to alleviate or to cure allergic diseases [1–3]. However, although SIT has been employed in the clinic for many years, the incidence of allergic diseases keeps rising [4]; this reflects that most of the allergic patients may not be able to reach SIT worldwide [5].

Bregs are one of the immune regulatory cell fractions. By producing interleukin- (IL-) 10 or/and transforming growth factor- (TGF-) beta, Bregs suppress other immune cells' activities that plays a critical role in maintaining the immune homeostasis in the body [6]. A portion of Bregs can be activated upon exposing to specific allergens, that is, designated allergen-specific Bregs (sBregs). It is reported that the Breg amounts are fewer or Bregs are dysfunctional in subjects with allergic disorders [6, 7]. Restoring Bregs renders the disease recovery [6, 7]. Yet, the underlying mechanism of maintaining Bregs at functional status is not fully understood yet; factors causing Breg dysfunction are to be further elucidated.

Published data indicate that oxidative stress is associated with the immune deregulation; for example, the oxidative stress-related inflammatory cytokine production is correlated with chronic kidney disease [8]. Oxidative stress is a condition that the amounts of oxidative species are beyond the capacity of antioxidants, such as superoxide dismutase (SOD), in the tissues [9]. The production of oxidative species is a physiological phenomenon in the tissues. These substances can be eliminated by the antioxidants in the tissues in general [9]. During allergy attacks, large amounts of oxidative species are produced in the local tissues that may be beyond the capacity of antioxidants and thus induce disorders in cells of the local tissues [10]. However, how Bregs are affected by oxidative species in an allergic environment remains to be further investigated.

It is known that flagellin (FGN) has immune regulatory functions [11]. FGN is the major component of the bacterial flagella. Published data indicate that FGN dichotomously involves in oxidative stress by counteracting or promoting oxidative stress depending on the cell activity profiles or/and the cytokine environment [11, 12]. Based on the information above, we hypothesize that FGN can promote the SIT efficacy by regulating Breg activities. To this end, we isolated Bregs from blood samples collected from patients with food allergy (FA) and healthy subjects. The role of FGN in the stabilization of Bregs was investigated.

## 2. Materials and Methods

### 2.1. Reagents

SOD1 inhibitor (ATN224, 15 *μ*M for cell culture; 4 mg/kg i.p. for mouse model study) was purchased from Baiolibo Inc. (Beijing, China). p38 inhibitor SB203580 was obtained from Calbiochem (San Diego, CA). Indicaxanthin was purchased from Molbase Biotech (Shanghai, China). Antibodies (Abs) of SOD1 (SOD, in short; clone# B-1), CD5 (UCH-T2, AF546), FasL (Kay-10), CD20 (D-10), STAT3 (F-2), pSTAT3 (B-7), CD19 (B-1, AF488), and IL-10 (3C12C12, AF647) were purchased from Santa Cruz Biotech (Santa Cruz, CA). Abs of pJNK (T183), NF-*κ*B (E39), pNF-*κ*B (EP2294Y), p38 (9F12), and p-p38 (EPR269Y) were purchased from Abcam (Cambridge, MA). ELISA kits of ROS, SOD, IL-4, IL-5, IL-13, IL-10, tryptase, mMCP1, ECP, and OVA-specific IgE were purchased from Biocompare (South San Francisco, CA). GL7 Ab (APC) was purchased from Dakewe BioMart (Beijing, China). Annexin V kit, OVA, and FGN were purchased from Sigma-Aldrich (St. Louis, MO). Extracts of food allergens (cow's milk, egg white, egg yolk, wheat flour, soybean, carrot, potato, and peanut) were purchased from Allergopharma (Germany).

### 2.2. Human Subjects

Patients with food allergy (FA) and healthy subjects were recruited into the present study at the Allergy Clinic of Longgang ENT Hospital and Longgang Central Hospital from June 2019 to August 2020. FA diagnosis was carried out by our physicians following our routine procedures, including food allergy history, serum-specific IgE positive, and food allergen skin prick test (SPT) positive. Patient selection was based on one of the main factors that affect SIT efficacy. The experimental procedures were approved by the Human Ethical Committee at Longgang Central Hospital (HECLC20190023) and Longgang ENT Hospital (HE20190035). A written informed consent was obtained from each human subject. The demographic data of human subjects are presented in Table 1. Human subject recruitment is also presented in a flow chart (Fig. S1 in supplemental materials). SPT results are presented in Fig. S2. Human sample experimental design is presented in Fig. S3. Subjects with any of the following conditions were excluded: autoimmune diseases, cancer, severe organ diseases, and in the treatment with immune suppressors or corticosteroids with any reasons.

### 2.3. Skin Prick Tests (SPTs)

SPT was performed in all FA patients. Allergens used in SPT included common aeroallergens, including mite mix, *Dermatophagoides farinae*, *Dermatophagoides pteronyssinus*, mold mix, pollens (Bermuda grass, pine, poplar, rye, timothy grass, and mugwort), animal dander (dog and cat), and food allergens (cow's milk, egg white, egg yolk, wheat flour, soybean, carrot, potato, and peanut). Allergenic extracts were purchased from Allergopharma (Germany). Histamine (10 mg/ml) and saline were used as positive and negative controls in SPT, respectively. If mean wheal diameter was ≥3 mm larger than the negative control, SPTs were considered positive.

### 2.4. Cell Culture

Cells were collected from relevant experiments and cultured in RPMI1640 medium supplemented with glutamine (2 mM), antibiotics (100 U/ml penicillin and 0.1 mg/ml streptomycin), and fetal calf serum (10%). The cell viability was greater than 99% as assessed by the Trypan blue exclusion assay.

### 2.5. Flow Cytometry (FACS)

Single cells were prepared in relevant experiments and incubated with 2% bovine serum albumin (BSA) for 30 min to block the nonspecific binding. In the surface staining, cells were stained with Abs of interest (labeled with fluorescence, diluted in 1 : 100) or isotype IgG for 30 min at 4°C. After washing with phosphate-buffered saline (PBS) 3 times, cells were analyzed with a flow cytometer (FACSCanto II, BD Bioscience). In the intracellular staining, cells were fixed with 1% paraformaldehyde for 1 h, washed with PBS 3 times, and followed by the procedures of the surface staining. The results were processed with a software package, FlowJo (TreeStar Inc., Ashland, OR), with the data obtained from the isotype IgG staining as gating controls.

### 2.6. Immune Cell Isolation

Single cells were prepared in relevant experiments and stained with fluorescence-labeled Abs. Targeted cells (detailed in figures) were sorted by FACS. Cell purity was checked by FACS. If purity did not reach 95%, the isolation was repeated.

### 2.7. Detection of Cell Apoptosis

Cells were collected from relevant experiments and stained with propidium iodide (PI) and Annexin V reagents following the manufacturer's instructions. The cells were analyzed by FACS. Annexin V^+^ cells or PI^+^ Annexin V^+^ cells were regarded as apoptotic cells.

### 2.8. Enzyme-Linked Immunosorbent Assay (ELISA)

The serum levels of cytokines (detailed in figures) were determined by ELISA using commercial reagent kits following the manufacturer's instructions.

### 2.9. Real-Time Quantitative RT-PCR (RT-qPCR)

Total RNA was extracted from cells collected from relevant experiments and converted to cDNA with a reverse transcription kit following the manufacturer's instructions. The cDNA samples were amplified in a qPCR device with the SYBR Green Master Mix and the presence of SOD primers (catcagtatggggacaatac and accagtgcaggacctcatttta). The results were calculated with the 2^-∆∆Ct^ method. The results were presented as the relative expression against the housekeeping gene *β*-actin.

### 2.10. Protein Extracts

Proteins were extracted from cells collected from relevant experiments by incubating with a lysis buffer for 30 min. Supernatant was collected by centrifuging the samples at 13,000 × g for 10 min. All the procedures were performed at 4°C.

### 2.11. Western Blotting

Protein extracts were prepared as described above, fractioned by SDS-PAGE and transferred onto a PVDF membrane. After incubating with skim milk (5%) for 30 min, the membrane was stained with Abs of interest (diluted in 1 : 500; the Ab types are detailed in relevant figures) overnight, washed with TBST (Tris-buffered saline mixed with Tween 20 at 0.05%) 3 times, followed by incubating with horseradish peroxidase- (HRP-) conjugated second Abs (diluted in 1 : 5,000) for 2 h, and washed with TBST 3 times. Immunoblots on the membrane were developed by the enhanced chemiluminescence and photographed in an imaging device (UVP, Cambridge, UK).

### 2.12. Mice

BALB/c mice (6-8-week-old) were purchased from Guangdong Experimental Animal Center (Guangzhou, China). Mice were maintained in a specific pathogen free facility with accessing food and water freely. The experimental procedures were approved by the Animal Ethical Committee at Shenzhen University.

### 2.13. RNA Sequencing (RNAseq)

sBregs were isolated from LPMCs by FACS. Total RNA was extracted from sBregs with a TRIzol reagent kit following the manufacturer instruction. RNA samples were analyzed by the staff of the YiGene Biotech company; RNAseq data were generated using the Illumina standard library preparation using an Illumina HiSeq 2500 sequencer, and an RNA library kit (TruSeq RNA library prep kit v2; Illumina) followed reported procedures [13]. RNAseq read quality was assessed using FastQC. The Beyers method was used to determine the differentially expressed genes (DEGs) [14]. Using htseq-count and DESeq [15], transcript counts were calculated and normalized. The log-transforming gene activities were reported as a heatmap and individual violin plots.

### 2.14. Collection of Gut Lavage Fluids (GLF)

Immediately from the stomach, a segment of small intestine (20 cm) was excised from mice. The intestinal cavity was rinsed with 3 ml saline (in a syringe). The recovered GLF was analyzed by ELISA.

### 2.15. Enzyme-Linked Immunosorbent Assay (ELISA)

Cytokine levels in the serum and GLF, SOD, and ROS in sBreg extracts were determined by ELISA with commercial reagent kits following the manufacturer's instructions. Human serum-specific IgE was determined by ImmunoCap with commercial reagent kits following the manufacturer's instruction and our routine procedures.

### 2.16. FA Mouse Model Development and Allergen-Specific Immunotherapy (SIT)

BALB/c mice were immunized by back skin injected with ovalbumin (OVA, 100 *μ*g/mouse) mixed in 0.1 ml alum in day 1 and day 7, respectively. Mice were boosted with gavage-feeding OVA (100 *μ*g/mouse) in 0.3 ml saline on days 9, 11, and 13, respectively. The mice were designated FA mice. Control mice were treated with saline in the same time points of FA mice. Following established procedures [16], FA mice were treated with oral SIT or/and FGN (2 *μ*g/mouse in 0.1 ml saline, i.p.). Briefly, the OVA was gavage-fed with the doses of 1 mg (days 15 and 16), 5 mg (days 18 and 19), 10 mg (days 20 and 21), 25 mg (days 23 and 24), and 50 mg (days 25-29). Control mice were treated with saline. On day 30, mice were challenged by gavage-feeding with OVA (50 mg/mouse) in 0.3 ml saline and followed by assessing the FA response with established procedures [16].

### 2.17. B Cell Depletion

To deplete B cell in mice, one dose of anti-mouse CD20 or isotype-matched Ab (100 *μ*g; used as a control) was injected in 200 *μ*l saline via tail veins [17]. This ensures that the B cell-depletion status lasts from day 7 to day 57 postinjection [18].

### 2.18. Statistics

The data are presented as mean ± SEM or median (IQR). The difference between the two groups was determined by the Student *t*-test on the normally distributed data or Mann-Whitney test if the data are nonnormally distributed. Multiple comparisons were performed with ANOVA followed by Dunnett's test or Bonferroni test. The correlation between two data sets was analyzed with Pearson coefficient test or Spearman's coefficient test. *P* < 0.05 was set as a significant criterion.

## 3. Results

### 3.1. Peripheral Bregs from FA Patients Show Lower TLR5 Activities and Higher Apoptotic Activities

Prompted by previous studies that show CD5^+^ B cells express IL-10 and have immune regulatory properties [19, 20], blood samples were collected from 30 FA patients and 30 healthy control (HC) subjects. Peripheral blood mononuclear cells (PBMCs) were isolated and analyzed by fluorescence-activated cell sorting (FACS). We found the lower CD5^+^ B cell frequency in FA patients than that in HC subjects. More than 90% CD5^+^ B cells showed IL-10^+^ (Fig. S4). CD5^+^ B cells showed immune suppressive effects on CD4^+^ T cell proliferation (Fig. S5). Thus, CD5^+^ B cells were called regulatory B cells (Breg) in this paper.

**Figure 1:**
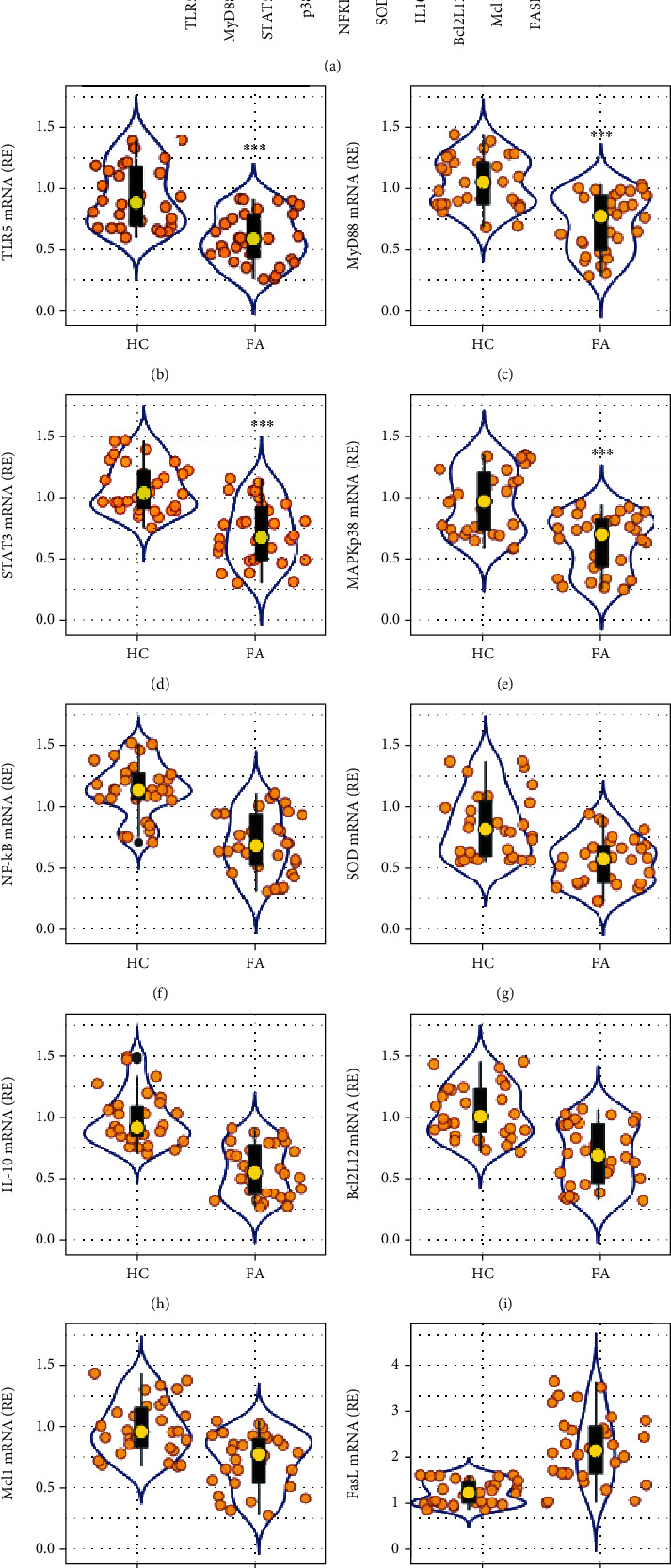
Assessment of DEGs in peripheral Bregs. CD19^+^ CD5^+^ Bregs were isolated from blood samples of 30 food allergy (FA) patients and 30 healthy control (HC) subjects and analyzed by RNAseq and RT-qPCR. (a) Heatmap shows the most active 10 DEGs in Bregs. (b) Violin plots show RT-qPCR results of the 10 DEGs in Bregs. The data of violin plots are presented as median (IQR). ^∗∗∗^*P* < 0.001 (Mann-Whitney test), compared with the HC group. Each bubble in violin plots shows data obtained from one sample.

Bregs were then isolated from PBMCs of both HC subjects and FA patients by magnetic cell sorting and analyzed by RNA sequencing (RNAseq).  Figure 1(a) presents the 10 most active differentiating expressing genes (DEGs) of RNAseq results, including lower TLR5 and its downstream signal components, oxidative-related superoxide dismutase (SOD), IL-10, apoptosis inhibitors (Bcl2L12 and Mcl-1), and higher FasL as compared with that in HC Bregs (Figure 1(a)). The RNAseq results were verified by conventional RT-qPCR assay with the same batch RNA samples used in RNAseq (Figures 1(b)–1(k)). The results implicate a link between the lower TLR5 activities and the lower Breg frequency in FA patients.

### 3.2. Peripheral Bregs Negatively Correlate with Serum FGN Levels in FA Patients

**Figure 2:**
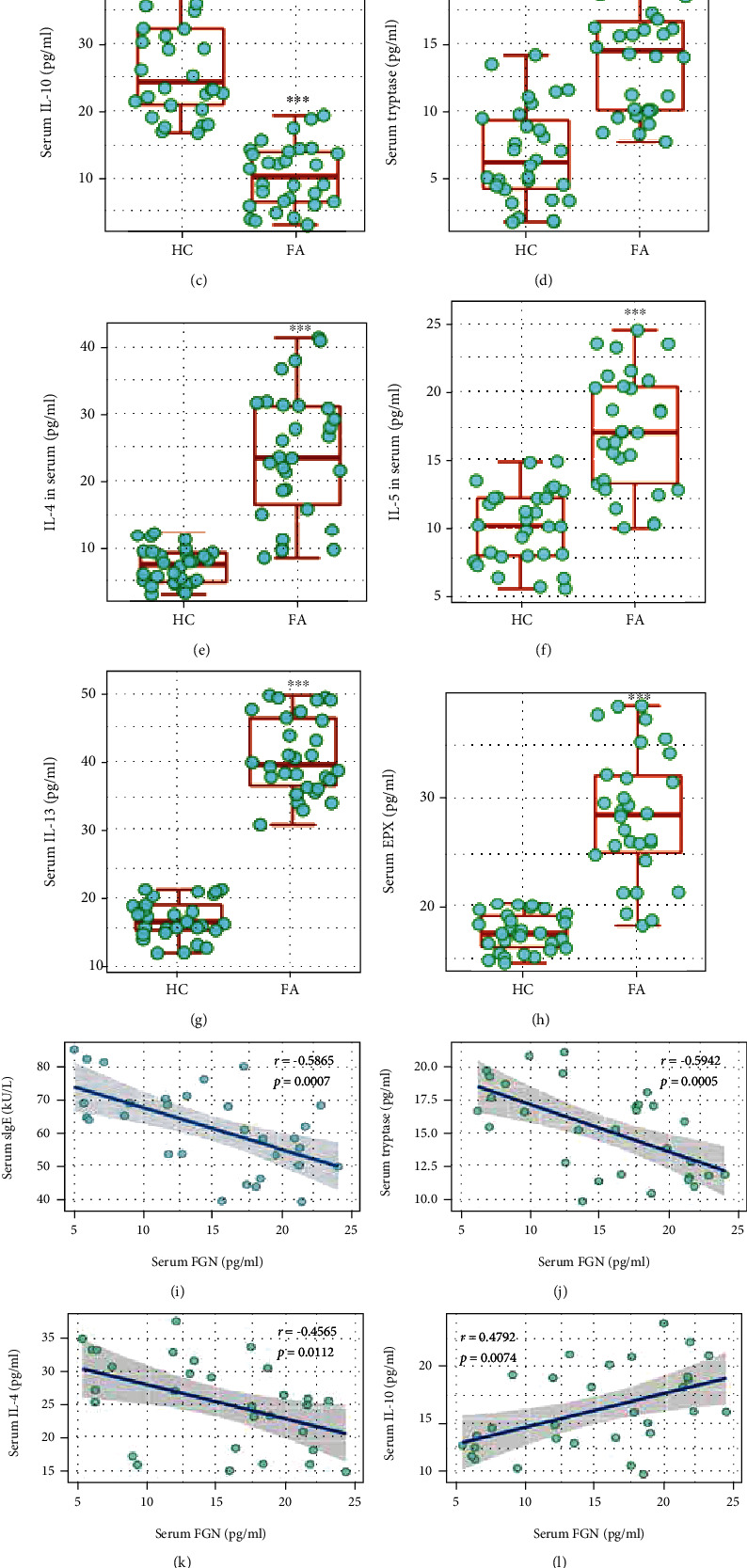
Assessment of association between serum FGN levels and immune response. Blood samples were obtained from FA patients (*n* = 30) and HC subjects (*n* = 30). The serum was isolated from blood samples and analyzed by ELISA. (a–h) Boxplots show serum levels of (a) specific IgE (sIgE), (b) FGN, (c) IL-10, (d) tryptase, (e–g) Th2 cytokines, and (h) EPX. (i–k) Negative correlation between serum FGN and serum levels of (i) sIgE, (j) tryptase, and (k) IL-4. (l–n) Positive correlation between (l) serum FGN and serum IL-10, (m) SOD mRNA in Bregs, and (n) peripheral Breg frequency. ^∗∗∗^*P* < 0.001 (Mann-Whitney test), compared with the HC group. The Spearman correlation coefficient test was performed in correlation assay. Each bubble in boxplots presents data obtained from one sample.

Prompted by the data of Figure 1 that show the lower TLR5 signal activities in Bregs, we examined flagellin (FGN) levels in the serum as FGN is the ligand of TLR5. Blood samples were collected from FA patients (*n* = 30) and HC subjects (*n* = 30). Serum samples were prepared and analyzed by ELISA. The results showed higher specific IgE (sIgE) levels in the serum (Figure 2(a)). FGN was detectable in the serum that was lower in FA samples than that in HC samples (Figure 2(b)). Notably, lower IL-10 levels and higher serum levels of tryptase, Th2 cytokines, and eosinophil peroxidase (EPX) were detected in FA samples than that in HC samples (Figures 2(b)–2(h)). Negative correlation was detected between serum FGN and serum sIgE, tryptase, or IL-4 (Figures 2(i)–2(k)). Positive correlation was detected between serum FGN and serum IL-10, IL-10 mRNA levels in Bregs, or peripheral Breg frequency (Figures 2(l)–2(n)). The results implicate a link between serum FGN levels and the aberrant Th2 response or/and Breg activities.

### 3.3. FGN Promotes Breg Survival

**Figure 3:**
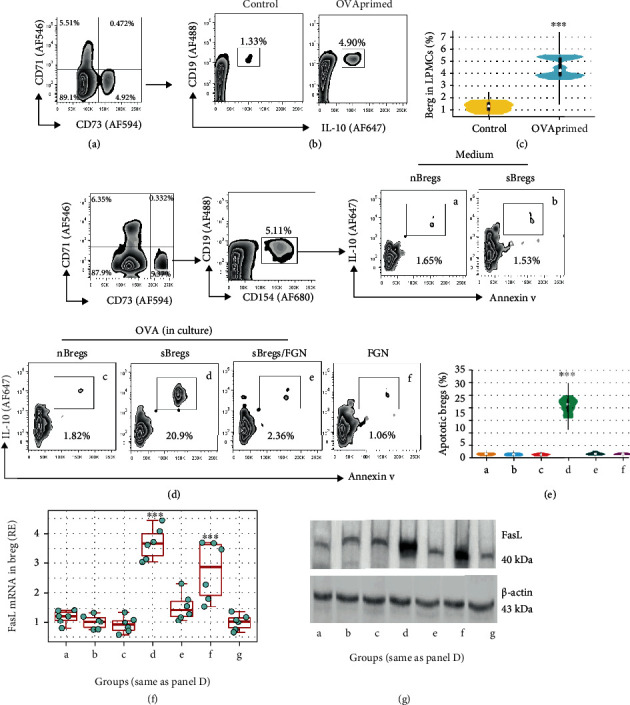
FGN counteracts the specific antigen-induced Breg apoptosis. LPMCs were prepared from naïve BALB/c mice or mice treated by gavage-feeding with OVA daily for one week and exposed to OVA (or BSA) in the culture overnight. (a) LPMCs were analyzed by FACS. Gated plots show GL7^+^ B cells (activated B cells). (b) Gated plots show CD5^+^ IL-10^+^ cells in the gated cells in panel Ad. (c) Boxplots show activated (by OVA) B cell frequency. (d–g) CD5^+^ B cells were isolated from LPMCs prepared naïve mice and OVA-primed mice and cultured in the presence of OVA (or BSA) overnight. Cells were stained with propidium iodide and Annexin V reagent and analyzed by FACS. Gated plots show (d) apoptotic cells. Boxplots show apoptotic (e) Breg frequency or (f) FasL mRNA in Breg. Immunoblots show (g) FasL protein levels in Bregs. The data of boxplots are presented as median (IQR). ^∗∗∗^*P* < 0.001 (ANOVA+Dunnett's test), compared with group a. Each bubble in boxplots presents data obtained from one sample.

Next, we fed naïve mice with a large dose of OVA (5 mg/mouse) daily for one week; this markedly induced Bregs (CD19^+^ CD5^+^ IL-10^+^) in the intestinal tissues, which also showed high expression of GL7 (an activation marker of lymphocytes) upon exposure to specific antigen, OVA, in culture overnight (Figures 3(a)–3(c)). The activation-induced cell death (AICD) was originally found in T cells [21]. It was latterly revealed that AICD was inducible in other cells, such as cancer cells [22]. We also found that activated by exposure to specific antigen induced Breg apoptosis, the FasL expression was increased in Bregs upon the specific antigen exposure, which could be counteracted by the presence of FGN (Figures 3(d)–3(g)). The data indicate that exposure to specific antigens can activate antigen-specific Bregs and subsequently induce Breg apoptosis, which can be counteracted by the presence of FGN.

### 3.4. FGN Diminishes Specific Antigen-Induced Oxidative Stress in Antigen-Specific Bregs (sBregs)

**Figure 4:**
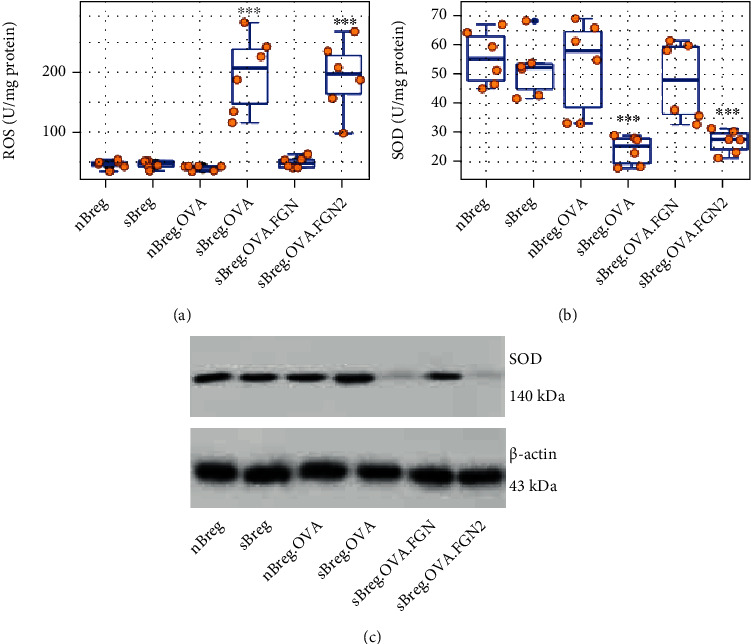
Exposure to specific antigen induces oxidative stress in sBregs. LPMCs were prepared from mice primed with OVA and were exposed to specific antigen, OVA, or and FGN, in the culture for 24 h. Naïve Bregs (nBregs) and sBregs were isolated by FACS. ROS and SOD in Breg cell extracts were assessed by ELISA. Boxplots show levels of (a) ROS and (b) SOD in Bregs. (c) Immunoblots show SOD protein in sBregs. ^∗∗∗^*P* < 0.001 (ANOVA followed by the Dunnett's test), compared with nBreg alone. Each bubble in boxplots presents data obtained from one experiment. FGN2: FGN+SOS inhibitor (ATN220; 1.5 *μ*M).

It is recognized that oxidative stress can be induced by immune response [23]. Reactive oxygen species (ROS) and superoxide dismutase (SOD) are the canonical factors in oxidative stress. To elucidate if exposure to specific antigen initiates oxidative stress in sBregs, LPMCs were isolated from OVA-primed mice and control mice and cultured in the presence or absence of OVA for 24 h, from which GL7^+^ CD5^+^ sBregs were purified by FACS and analyzed by ELISA. We found that, upon exposure to a specific antigen (OVA), ROS levels were markedly increased in sBregs, and the SOD levels were markedly decreased in sBregs, but not in naïve Bregs (nBregs). The presence of FGN in culture attenuated the changes of ROS and SOD in Bregs induced by antigen exposure that was abolished in the presence of ATN220 (an SOD inhibitor) (Figure 4). The results indicate that exposure to specific antigens induces oxidative stress in sBregs that can be counteracted by FGN, and the latter promotes SOD expression in sBregs.

### 3.5. The STAT3/MAPKp38/NF-*κ*B Pathway Is Involved in FGN/TLR5 Signal-Promoted SOD Expression in Bregs

**Figure 5:**
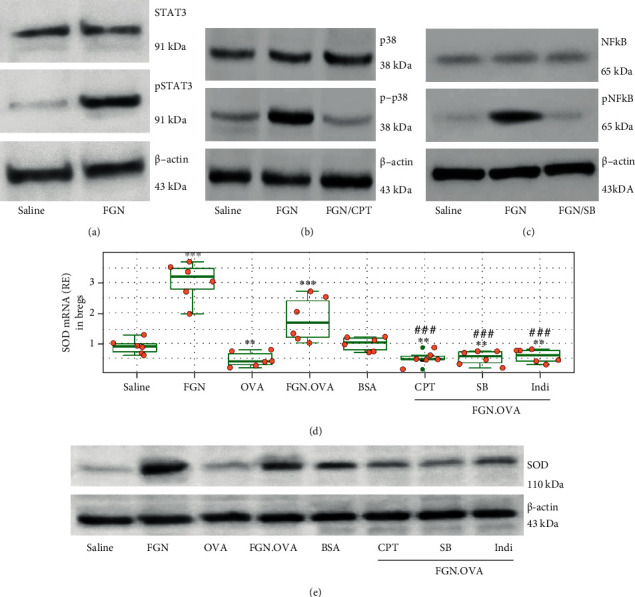
Signal pathway of FGN upregulating SOD expression in sBregs. LPMCs were isolated from OVA-primed mouse intestine and cultured in the conditions denoted below (e). (a–c) Immunoblots show (a) STAT3/pSTAT3, (b) p38/p-p38, and (c) NF-*κ*B/pNF-*κ*B in sBregs. (d) Boxplots and (e) immunoblots show SOD levels in sBregs. FGN: 1 *μ*g/ml; OVA: 5 *μ*g/ml; BSA: 5 *μ*g/ml; CPT: Cryptotanshinone (a STAT3 inhibitor, 5 *μ*M); SB: SP203580 (a p38 inhibitor, 10 *μ*M); Indi: indicaxanthin (an NF-*κ*B inhibitor, 20 *μ*M). The data of boxplots are presented as median (IQR). The data represent 3 independent experiments.

As FGN activates STAT3 [24] promotes MAPKp38's activity [25], and NF-*κ*B is associated with SOD gene transcription [26], we inferred that the STAT3/MAPKp38/NF-*κ*B might be the signal pathway through which FGN u regulated the SOD expression in sBregs. Therefore, we tested the role of FGN in activating the STAT3/MAPKp38/NF-*κ*B pathway in sBregs. Mice were primed by feeding OVA daily for one week to generate Bregs in the intestine. LPMCs were isolated and cultured in the presence of OVA or/and FGN with or without the addition of the STAT3 inhibitor (Cryptotanshinone or CPT) or MAPKp38 inhibitor (SP203580) or NF-*κ*B inhibitor (indicaxanthin), respectively. CD19^+^ CD5^+^ GL7^+^ sBregs were purified from the LPMCs by FACS. RNAs and proteins were extracted from nBregs and sBregs and analyzed by RT-qPCR and Western blotting. Firstly, we observed that FGN did activate the STAT3/MAPKp38/NF-*κ*B signal pathway (Figures 5(a)–5(c)). Exposure to OVA in culture markedly diminished the SOD expression in sBregs that was abrogated by the presence of FGN. The effects of FGN were abolished by the presence of STAT3 inhibitor or MAPKp38 inhibitor or NF-*κ*B inhibitor (Figures 5(e) and 5(f)). The results demonstrate that FGN upregulates the SOD expression in sBregs by activating the STAT3/MAPKp38/NF-*κ*B pathway. In addition, we also observed that FGN increased the SOD expression in nBregs (data not shown).

### 3.6. Administration of FGN Promotes SIT Effects on Experimental FA by Promoting Breg Survival

**Figure 6:**
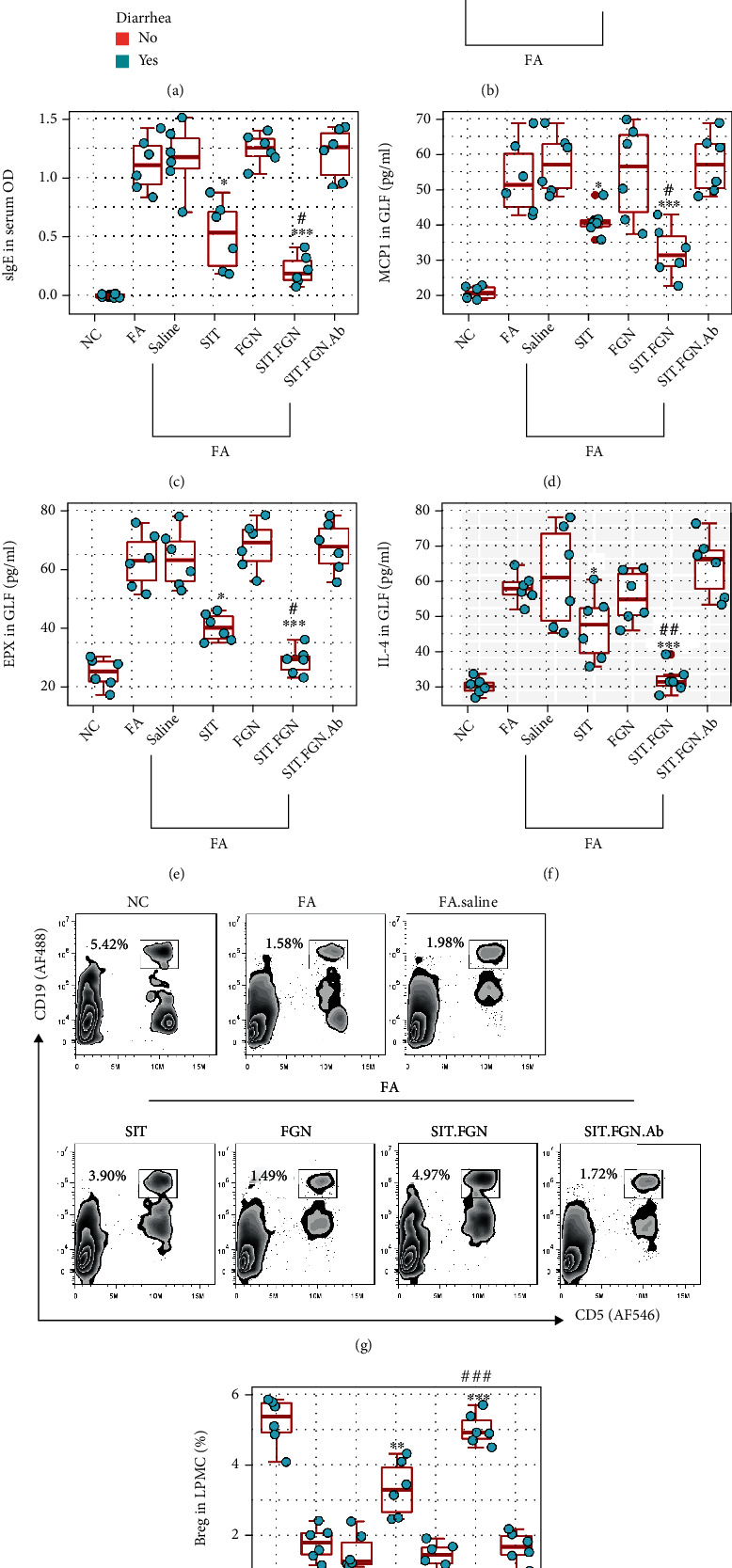
FGN promotes SIT efficacy in suppressing FA response in mice. A murine FA model was developed. FA mice were treated with the procedures as denoted in each panel. (a) Number of mice with diarrhea. (b) Core temperature (Temp) changes in mice recorded 30 min after the last antigen challenge. (c) Serum-specific IgE (sIgE) levels. (d–f) MCP1, EPX, and IL-4 levels in GLF (gut lavage fluids). (g, h) LPMCs were prepared and analyzed by FACS. (g) Gated plots show Breg counts. (h) Boxplots show Breg frequency in LPMCs. Data of boxplots are presented as median (IQR). ^∗^*P* < 0.05, ^∗∗∗^*P* < 0.01, and ^∗∗∗^*P* < 0.001 (ANOVA followed by Dunnett's test), compared with the FA group. ^#^*P* < 0.05, ^##^*P* < 0.01, and ^###^*P* < 0.001 (ANOVA+Dunnett's test), compared with the SIT group. Each group consists of 6 mice. Each bubble in boxplots presents data obtained from one mouse. NC: naïve control mice; FA: FA mice; saline: FA mice were treated with saline; SIT (FGN, SIT, and FGN): mice were treated with SIT (or FGN or FGN+SIT); Ab: mice were treated with anti-CD20 Ab to deplete B cells.

We then developed an FA mouse model (Fig. S6). In response to specific antigen challenge, FA mice showed diarrhea, drop of the core temperature, increase in serum specific IgE, and allergy-related cytokines, including MCP1, EPX, and IL-4, in gut lavage fluids (GLF). FA mice were treated with SIT or/and FGN in a two-week period (Fig. S6). As shown in Figure 6, SIT alone resulted appreciable suppressive effects on FA response that was promoted by the combination of SIT and FGN. Treating with FGN alone did not gain appreciable therapeutic effects. LPMCs were prepared from mice and analyzed by FACS. The results showed that the Breg frequency in LPMC was markedly increased in mice treated with both SIT and FGN, while those treated with SIT alone or FGN alone did not show appreciable increase in Bregs. The results were verified by a further experiment, in which depletion of Bregs by administration of anti-CD20 Ab [17] (Fig. S7) abolished the suppressive effects on FA response by combination of SIT and FGN (Figure 6).

## 4. Discussion

The present study revealed a novel phenomenon that administration of FGN promoted the SIT efficacy in experimental FA. We found that exposure to specific antigens induced Breg apoptosis. In response to specific antigens, the frequency of apoptotic sBregs was increased in the intestine, in which the oxidative stress was detected. Because we found lower FGN levels in the serum and lower TLR5 signals in Bregs of FA patient, FA Bregs were exposed to specific antigens and FGN concurrently; this prevented the Breg apoptosis. The presence of FGN could increase the SOD expression in Bregs; SOD then suppressed the specific antigen-induced oxidative stress in Bregs. Administration of FGN potentiated the SIT effects on suppressing FA response in mice by stabilizing sBregs in the intestine.

The data show that concomitant administration of FGN promotes SIT efficacy in experimental FA. SIT has been employed in the treatment of allergic diseases as it is regarded as a specific remedy which is aimed at cure allergic diseases [2]. By administering escalating small doses of specific antigens, it is expected to generate immune regulatory cells, such as Tregs and Bregs, in targeted subjects. This has been verified by cumulative studies [3]. For example, a retrospective clinical study, which observed allergic rhinitis patients receiving birch pollen SIT, revealed that up to 6 years of follow-up, significantly more SIT patients were found to be medication-free [27]. In milk allergy patients, significant improvement was gained in patients after SIT [28]. However, the therapeutic efficacy of SIT is variously reported. Roger et al. indicated that SIT was applied in a cohort of allergic rhinitis patients, and allergic parameters, including IFN-*γ*, IL-4, IL-5, IL-10, IL-13, and specific IgE, were not appreciably altered by SIT [29]. Glover et al. felt “uncertain” about the efficacy of SIT on asthma patients [30], while Galli et al. even found no difference in the allergic symptoms between the SIT group and the control group [31]. The above information indicates that improvement of the SIT efficacy is still needed. The present data show that concurrent administration of FGN and SIT can efficiently promote the SIT efficacy; this is expected to promote the SIT efficacy in the treatment of allergic diseases.

It is proposed that the therapeutic effects of SIT on allergic diseases are generated and maintained by the antigen-specific immune regulatory cells, such as Tregs and Bregs [7]. Yet, little inflammation about the destiny of SIT-generated Bregs is available in the literature. The present study provided information to this knowledge gap. The reexposure to specific antigens can induce Breg apoptosis. Reexposure to specific antigens occurs often in real life. For example, house dust mites that extensively distribute in the human living environment and can enter the body through inhaling or swallowing with contaminated food [32], which can be the source of antigens to induce Breg apoptosis to impair the immune regulatory functions. Although FA patients can avoid taking in the allergic food to prevent FA attacks, however, some peptides in food allergens are cross-expressed by more than one species [33]. SIT is to introduce small doses of specific antigens into the body. Besides the role of inducing Bregs, whether the introduced antigens cause existing Breg apoptosis in human is worth to be investigated.

We found that exposure to specific antigens induced Breg apoptosis. The underlying mechanism of this phenomenon may be that the specific antigens activate the antigen-specific Bregs. The activation initiates the apoptosis program in Bregs. This is supported by published data. The activation-induced T cell death (AICD) has been recognized for many years [34]. Our data also show that exposure to specific antigens can increase the activation of FasL in Bregs. FasL is a key initiator of apoptosis; it is also the canonical trigger in AICD [34]. The RNAseq data showed that SOD gene activities were diminished in Bregs of FA patients. SOD is one of the major antioxidants. Deregulation of SOD production is usually associated with immune diseases [35]. The data show that FGN upregulates SOD expression in Bregs, and abrogates the specific antigen-induced oxidative stress in Bregs and Breg apoptosis. Therefore, although high levels of FGN may cause disorders in the body [36], FGN at proper dosage can still be beneficial to the immune regulation by preventing the allergen exposure-induced Breg apoptosis, as demonstrated by the present study and others [11].

The data show that administration of FGN promotes the SIT efficacy to alleviate experimental FA. It is the consensus that SIT is a specific remedy for allergic diseases [27, 28]. Yet, the therapeutic efficacy of SIT needs to be improved. The present data show that a combination of SIT and FGN can promote the therapeutic effects on experimental FA through a mechanism to maintain Breg homeostasis by promoting Breg survival, implicating its application in human allergic disease treatment.

In summary, the present data show that exposure to specific antigens can induce Breg apoptosis through increasing oxidative stress in Bregs. Administration of FGN promotes therapeutic effects of SIT on suppressing experimental FA by promoting Breg survival. The data suggest that the combination of FGN and SIT can be a novel therapy that has translation potential in the treatment of allergic diseases.

## Figures and Tables

**Table 1:** Demographic data of human subjects.

Items	FA	HC
Female/male	14/16	15/15
Age (years)	18 (12-36)	21 (18-28)
Blood eosinophil	5.5% (4.1%-6.8%)	1.6% (1.1-3.5%)^∗^
Food allergen SPT+	30 (100%)	0
Food allergy history	30 (100%)	0
Asthma	3 (10%)	0
Allergic rhinitis	3 (10%)	0
Allergic dermatitis	1 (6.6%)	0
Smoking history	0	0

^∗^ *P* < 0.01, compared with the FA group.

## Data Availability

Data used to support the findings of this study are available from the corresponding author upon request.

## Abbreviations

SIT: Allergen-specific immunotherapy
FGN: Flagellin
FA: Food allergy
HC: Healthy control
Bregs: Regulatory B cells
Tregs: Regulatory T cells
TGF: Transforming growth factor
IL: Interleukin
sBregs: Antigen-specific Bregs
SOD: Superoxide dismutase.

## Supplementary Materials

Supplementary Materials Figure S1: a flow chart shows human subject recruitment. Figure S2: skin prick test (SPT) results. SPT was performed for each FA subject. Bar plots show SPT results of food allergens (A) and air borne allergens (B). None of the healthy control subjects showed positive SPT results for these allergens (not shown). Figure S3: experimental design for human samples. Figure S4: assessment of CD5^+^ IL-10^+^ B cell frequency in PBMCs. Figure S5: assessment of immune suppressive effects of CD5^+^ B cells on T cell proliferation. Figure S6: procedures of an FA mouse model development and SIT. Figure S7: depletion of B cells in mice.

## References

[1] Moote W., Kim H., Ellis A. K. (2018). Allergen-specific immunotherapy. *Allergy, Asthma & Clinical Immunology*.

[2] Larché M., Akdis C. A., Valenta R. (2006). Immunological mechanisms of allergen-specific immunotherapy. *Nature Reviews. Immunology*.

[3] Głobińska A., Boonpiyathad T., Satitsuksanoa P. (2018). Mechanisms of allergen-specific immunotherapy: diverse mechanisms of immune tolerance to allergens. *Annals of Allergy, Asthma & Immunology*.

[4] Platts-Mills T. A. (2015). The allergy epidemics: 1870-2010. *The Journal of Allergy and Clinical Immunology*.

[5] Alvaro-Lozano M., Akdis C. A., Akdis M. (2020). EAACI allergen immunotherapy user's guide. *Pediatric Allergy and Immunology*.

[6] Rosser E. C., Mauri C. (2015). Regulatory B cells: origin, phenotype, and function. *Immunity*.

[7] Satitsuksanoa P., van de Veen W., Akdis M. (2019). B-cell responses in allergen immunotherapy. *Current Opinion in Allergy and Clinical Immunology*.

[8] Granata S., Dalla Gassa A., Bellin G., Lupo A., Zaza G. (2016). Transcriptomics: a step behind the comprehension of the polygenic influence on oxidative stress, immune deregulation, and mitochondrial dysfunction in chronic kidney disease. *BioMed Research International*.

[9] Sies H. (2015). Oxidative stress: a concept in redox biology and medicine. *Redox Biology*.

[10] Mishra V., Banga J., Silveyra P. (2018). Oxidative stress and cellular pathways of asthma and inflammation: therapeutic strategies and pharmacological targets. *Pharmacology & Therapeutics*.

[11] Tran H. Q., Ley R. E., Gewirtz A. T., Chassaing B. (2019). Flagellin-elicited adaptive immunity suppresses flagellated microbiota and vaccinates against chronic inflammatory diseases. *Nature Communications*.

[12] Liaudet L., Murthy K. G., Mabley J. G. (2002). Comparison of inflammation, organ damage, and oxidant stress induced by Salmonella enterica serovar Muenchen flagellin and serovar Enteritidis lipopolysaccharide. *Infection and Immunity*.

[13] Guo G., Sun X., Chen C. (2013). Whole-genome and whole-exome sequencing of bladder cancer identifies frequent alterations in genes involved in sister chromatid cohesion and segregation. *Nature Genetics*.

[14] Sartor M. A., Tomlinson C. R., Wesselkamper S. C., Sivaganesan S., Leikauf G. D., Medvedovic M. (2006). Intensity-based hierarchical Bayes method improves testing for differentially expressed genes in microarray experiments. *BMC Bioinformatics*.

[15] Anders S., Huber W. (2010). Differential expression analysis for sequence count data. *Genome Biology*.

[16] Zhang H. P., Wu Y., Liu J. (2013). TSP1-producing B cells show immune regulatory property and suppress allergy- related mucosal inflammation. *Scientific Reports*.

[17] Garnelo M., Tan A., Her Z. (2017). Interaction between tumour-infiltrating B cells and T cells controls the progression of hepatocellular carcinoma. *Gut*.

[18] Montalvao F., Garcia Z., Celli S. (2013). The mechanism of anti-CD20-mediated B cell depletion revealed by intravital imaging. *The Journal of Clinical Investigation*.

[19] Noh J., Noh G., Kim H. S., Kim A. R., Choi W. S. (2012). Allergen-specific responses of CD19(+)CD5(+)Foxp3(+) regulatory B cells (Bregs) and CD4(+)Foxp3(+) regulatory T cell (Tregs) in immune tolerance of cow milk allergy of late eczematous reactions. *Cellular Immunology*.

[20] Lee J. H., Noh J., Noh G. (2010). Allergen-specific B cell subset responses in cow's milk allergy of late eczematous reactions in atopic dermatitis. *Cellular Immunology*.

[21] Zhang M., Ming S., Gong S. (2019). Activation-induced cell death of mucosal-associated invariant T cells is amplified by OX40 in type 2 diabetic patients. *Journal of Immunology*.

[22] Han S., Tie X., Meng L., Wang Y., Wu A. (2013). PMA and ionomycin induce glioblastoma cell death: activation-induced cell-death-like phenomena occur in glioma cells. *PLoS One*.

[23] Tian T., Wang Z., Zhang J. (2017). Pathomechanisms of oxidative stress in inflammatory bowel disease and potential antioxidant therapies. *Oxidative Medicine and Cellular Longevity*.

[24] Yu Y., Zeng H., Vijay-Kumar M. (2004). STAT signaling underlies difference between flagellin-induced and tumor necrosis factor-alpha-induced epithelial gene expression. *The Journal of Biological Chemistry*.

[25] Illek B., Fu Z., Schwarzer C. (2008). Flagellin-stimulated Cl- secretion and innate immune responses in airway epithelia: role for p38. *American Journal of Physiology. Lung Cellular and Molecular Physiology*.

[26] Sugino N., Karube-Harada A., Sakata A., Takiguchi S., Kato H. (2002). Nuclear factor-kappa B is required for tumor necrosis factor-alpha-induced manganese superoxide dismutase expression in human endometrial stromal cells. *The Journal of Clinical Endocrinology and Metabolism*.

[27] Wahn U., Bachert C., Heinrich J., Richter H., Zielen S. (2019). Real-world benefits of allergen immunotherapy for birch pollen-associated allergic rhinitis and asthma. *Allergy*.

[28] Taniuchi S., Takahashi M., Soejima K., Hatano Y., Minami H. (2017). Immunotherapy for cow's milk allergy. *Human Vaccines & Immunotherapeutics*.

[29] Roger A., Depreux N., Jurgens Y., Heath M. D., Garcia G., Skinner M. A. (2014). A novel and well tolerated mite allergoid subcutaneous immunotherapy: evidence of clinical and immunologic efficacy. *Immunity, Inflammation and Disease*.

[30] Glover M. T., Atherton D. J. (1992). A double-blind controlled trial of hyposensitization to Dermatophagoides pteronyssinus in children with atopic eczema. *Clinical and Experimental Allergy*.

[31] Galli E., Chini L., Nardi S. (1994). Use of a specific oral hyposensitization therapy to Dermatophagoides pteronyssinus in children with atopic dermatitis. *Allergol Immunopathol*.

[32] Tulic M. K., Vivinus-Nébot M., Rekima A. (2016). Presence of commensal house dust mite allergen in human gastrointestinal tract: a potential contributor to intestinal barrier dysfunction. *Gut*.

[33] Valenta R., Hochwallner H., Linhart B., Pahr S. (2015). Food allergies: the basics. *Gastroenterology*.

[34] Green D. R., Droin N., Pinkoski M. (2003). Activation-induced cell death in T cells. *Immunological Reviews*.

[35] Yahfoufi N., Alsadi N., Jambi M., Matar C. (2018). The immunomodulatory and anti-inflammatory role of polyphenols. *Nutrients*.

[36] Markazi A., Bracci P. M., McGrath M., Gao S. J. (2020). Pseudomonas aeruginosa stimulates inflammation and enhances Kaposi's sarcoma herpesvirus-induced cell proliferation and cellular transformation through both lipopolysaccharide and flagellin. *Mbio*.

