# Supplementary material for: Modulating Oxidative Stress in B Cells Promotes Immunotherapy in Food Allergy
Source: Oxid Med Cell Longev. 2022 Jan 21;2022:3605977. doi: 10.1155/2022/3605977 (PMC8799367; doi:10.1155/2022/3605977)
Supplement: Supplementary Materials — Figure S1: a flow chart shows human subject recruitment. Figure S2: skin prick test (SPT) results. SPT was performed for each FA subject. Bar plots show SPT results of food allergens (A) and air borne allergens (B). None of the healthy control subjects showed positive SPT results for these allergens (not shown). Figure S3: experimental design for human samples. Figure S4: assessment of CD5+ IL-10+ B cell frequency in PBMCs. Figure S5: assessment of immune suppressive effects of CD5+ B cells on T cell proliferation. Figure S6: procedures of an FA mouse model development and SIT. Figure S7: depletion of B cells in mice. [file 3605977.f1.docx]

**Supplemental materials**


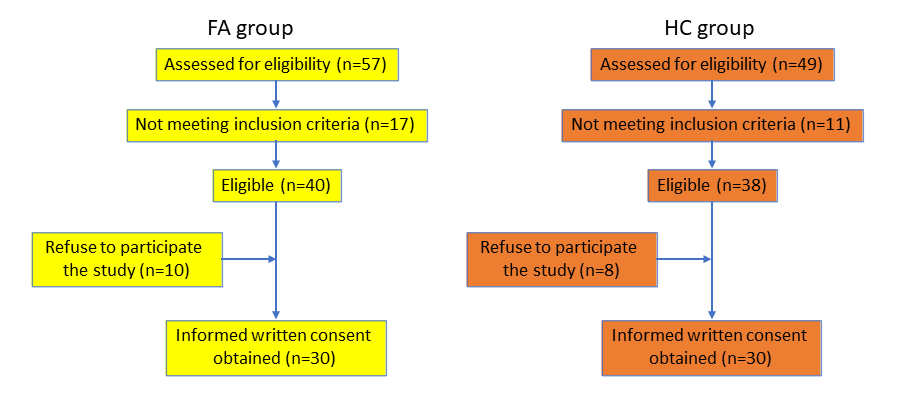


**Figure S1. A flow chart shows human subject recruitment**.


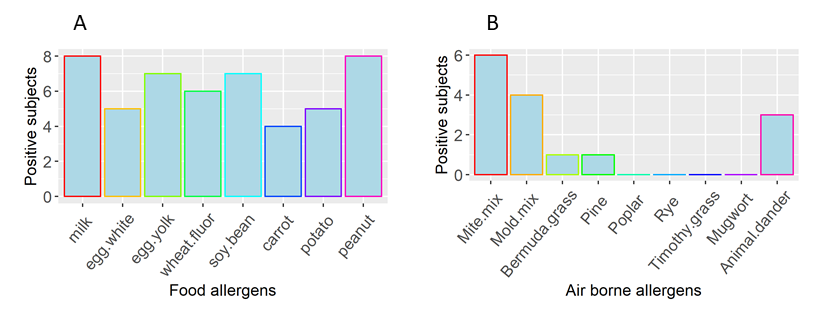


**Figure S2. Skin prick test (SPT) results**. SPT was performed for each FA subject. Bar plots show SPT results of food allergens (A) and air borne allergens (B). None of the healthy control subjects showed positive SPT results for these allergens (not shown).


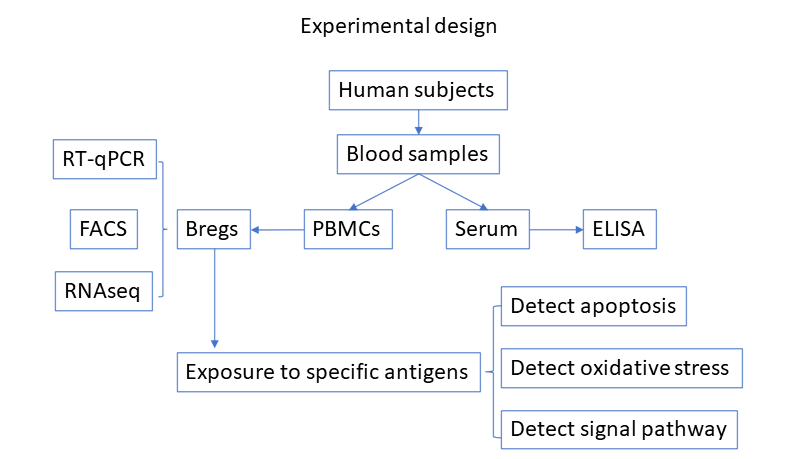


**Figure S3. Experimental design for human samples**.


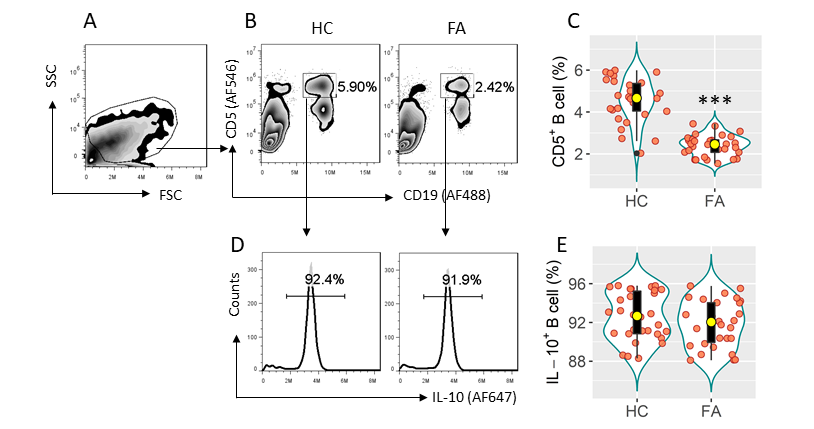


**Figure S4. Assessment of CD5^+^ IL-10^+^ B cell frequency in PBMCs**. PBMCs were prepared from blood samples obtained from 30 HC subjects and 30 FA subjects, and analyzed by FACS. A, the FSC and SSC gating plots. B, gated FACS plots show CD5^+^ CD19^+^ B cells in PBMCs. C, violin plots show CD5^+^ CD19^+^ B cell frequency. D, gated histograms show IL-10^+^ cells in CD5^+^ B cells. E, violin plots show IL-10^+^ CD5^+^ B cell frequency. The presented FACS data are from one experiment that represent 30 independent experiments. Data of violin plots are presented as median (IQR). Each bubble in violin plots presents data obtained from one sample. ***, p<0.001 (Mann Whitney test), compared with the HC group.


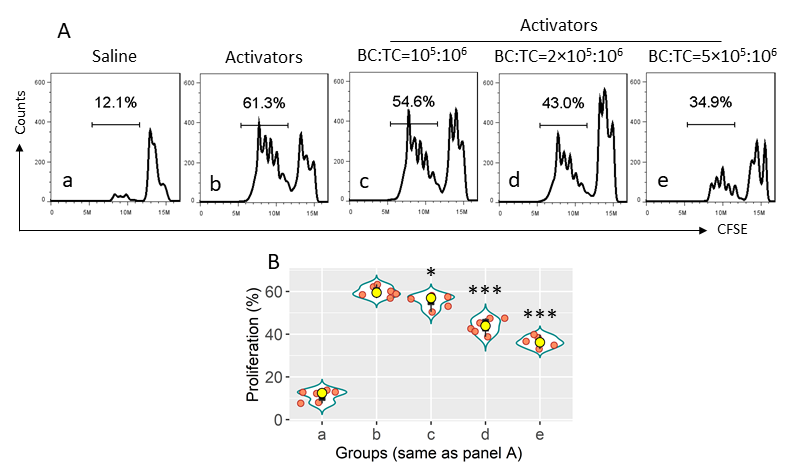


**Figure S5. Assessment of immune suppressive effects of CD5^+^ B cells on T cell proliferation**. PBMCs were isolated from HC subjects, from which CD5^+^ B cells (BC) and CD4^+^ CD25⁻ T cells (TC, labeled with CFSE) were isolated by magnetic cell sorting. BC and TC were co-cultured in the conditions denoted above each subpanel. Activators: Anti-CD3 (2 µg/ml)/CD28 (5 µg/ml) Abs for TC activation; LPS (100 ng/ml) for BC activation. A, gated histograms show proliferating TC. B, violin plots show proliferating TC frequency. The data of violin plots are presented as median (IQR). *, p<0.05, ***, p<0.001 (ANOVA + Dunnett’s test), compared with group b. Each bubble in violin plots presents data obtained from one sample.


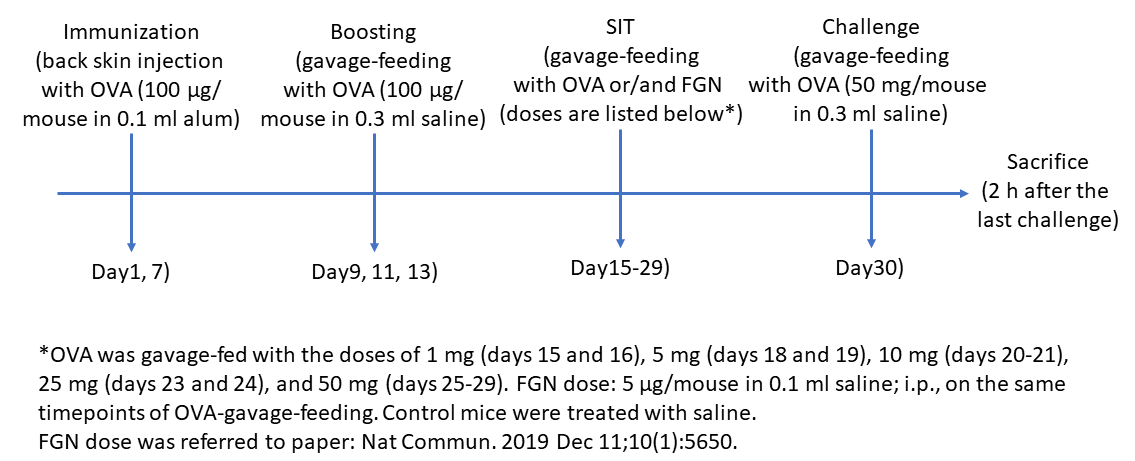


**Figure S6. Procedures of an FA mouse model development and SIT**.


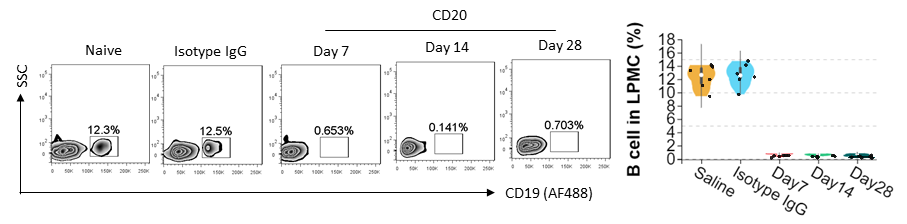


**Figure S7. Depletion of B cells in mice**. Mice were injected with anti-CD20 Ab (0.1 mg/mouse in 0.1 ml saline) or isotype IgG through the tail vein. Mice were sacrificed on day 7, or day 14, or day 28 post-injection. LPMCs were prepared from mice upon the sacrifice, and analyzed by FACS. Gated plots show B cell frequency. Violin plots show summarized B cell frequency in LPMCs. Each dot in violin plots presents data obtained from one mouse.
